# Genetics of Major Depressive Disorder in a Homogeneous Population with Uniform Phenotyping

**DOI:** 10.21203/rs.3.rs-6238738/v1

**Published:** 2025-10-31

**Authors:** Floris Huider, Yuri Milaneschi, René Pool, Bernardo de A.P.C. Maciel, Scott D. Gordon, M. Liset Rietman, Almar A.L. Kok, Tessel E. Galesloot, Brittany L. Mitchell, Leen M. ‘t Hart, Femke Rutters, Marieke T. Blom, Didi Rhebergen, Marjolein Visser, Ingeborg A. Brouwer, Edith Feskens, Catharina A. Hartman, Albertine J. Oldehinkel, Mariska Bot, Eco J.C. de Geus, Lambertus A. Kiemeney, Martijn Huisman, H. Susan J. Picavet, W.M. Monique Verschuren, Nicholas G. Martin, Conor Dolan, Hanna M. van Loo, Brenda W.J.H. Penninx, Jouke-Jan Hottenga, Dorret I. Boomsma

**Affiliations:** 1 Department of Complex Trait Genetics, Vrije Universiteit Amsterdam, 1081 Amsterdam, The Netherlands.; 2 Amsterdam Public Health Research Institute, Amsterdam, The Netherlands.; 3 Department of Biological Psychology, Faculty of Behavioral and Movement Sciences, Vrije Universiteit Amsterdam, 1081 Amsterdam, The Netherlands.; 4 Department of Psychiatry, Amsterdam UMC location Vrije Universiteit Amsterdam, 1081 Amsterdam, The Netherlands.; 5 Amsterdam Neuroscience, Complex Trait Genetics, 1081 Amsterdam, The Netherlands.; 6 Brain and Mental Health Program, QIMR Berghofer Medical Research Institute, Brisbane, Australia.; 7 Center for Prevention, Lifestyle and Health, Dutch National Institute for Public Health and the Environment, 3721 Bilthoven, The Netherlands.; 8 Department of Epidemiology and Data Science, Amsterdam UMC, 1081 Amsterdam, The Netherlands.; 9 IQ Health Science Department, Radboud University Medical Center, 6525 Nijmegen, The Netherlands.; 10 Department of Cell and Chemical Biology, Leiden University Medical Center, 2333 Leiden, The Netherlands.; 11 Department of Biomedical Data Sciences, Section Molecular Epidemiology, Leiden University Medical Center, 2333 ZA Leiden, The Netherlands.; 12 Department of General Practice, Amsterdam UMC, 1081 Amsterdam, The Netherlands.; 13 Department of Health Sciences, Faculty of Science, Vrije Universiteit Amsterdam, 1081 Amsterdam, The Netherlands.; 14 Division of Human Nutrition and Health, Wageningen University & Research, 6700 Wageningen, The Netherlands.; 15 Department of Psychiatry, University of Groningen, University Medical Center Groningen, 9713 Groningen, The Netherlands.; 16 Department of Urology and Department for Health Evidence, Radboud University Medical Center, 6525 Nijmegen, The Netherlands; 17 Department of Sociology, Vrije Universiteit Amsterdam, 1081 Amsterdam, The Netherlands.; 18 Julius Center for Health Sciences and Primary Care, University Medical Center Utrecht, 3584 Utrecht, The Netherlands.

**Keywords:** GWAS, MDD, uniform population, DSM criteria, PGS

## Abstract

Harmonized phenotyping and diverse population-specific studies are crucial for advancing gene discovery in psychiatric genetics. We conducted a genome-wide association study (GWAS) of DSM-defined lifetime Major Depressive Disorder (MDD) in 64,941 participants (25.7% cases) from the Dutch BIObanks Netherlands Internet Collaboration (BIONIC) consortium. SNP-based heritability was estimated at 13.4%, exceeding recent global meta-analyses, with a high genetic correlation (r = 0.89) to the latest major depression GWAS by the Psychiatric Genetics Consortium (PGC-MD). We identified a novel genome-wide significant locus in PALMD (P = 3.26 × 10^−8^), that was confirmed by GWAS-by-subtraction. Polygenic scores (PGSs) based on BIONIC predicted MDD in UKBiobank, and PGSs from PGC-MD predicted into BIONIC, with within-family analyses indicating minimal confounding. Genetic causal inference revealed associations with over 30 phenotypes. Twin concordance for MDD increased with polygenic burden, reinforcing its genetic architecture. This study emphasizes the power of harmonized phenotyping and regional biobanks in uncovering the genetic architecture of MDD, highlighting the value of population-specific studies for improving risk prediction and advancing psychiatric genetics.

Major depressive disorder (MDD) is among the most common psychiatric disorders with a lifetime prevalence that can reach up to 19%, and is a leading cause of disability worldwide^1,2^. Episodes are frequently characterized by severe dysfunction and pervasiveness^3,4^. MDD is a genetically complex trait with a twin-based heritability of 37%^5^ and a polygenic architecture, characterized by the additive effect of many variants with small effects. In addition, MDD’s intrinsic heterogeneity, heterogeneity in depression diagnoses and assessment, and the relatively modest heritability compared to other psychiatric conditions, have all been suggested as contributing to low statistical power for genome-wide association studies^6,7^. One successful approach to combating several of these problems is through large international collaborative efforts that bring together dozens of cohorts and millions of individuals^8–11^.

At the same time, a discussion has emerged regarding a trade-off in sample size and phenotypic definition in genetic studies. Larger sample sizes have led to finding large numbers of genetic variants associated with broad concepts of depression. Growing sample sizes often come at the expense of uniform phenotyping; combining samples across multiple studies tends to yield a lower adherence to clinical diagnostic criteria and phenotypic uniformity. The use of self-reported diagnosis or treatment for depression, or depressed mood derived from a cut-off applied to a self-report symptom questionnaire in research, coined ‘minimal phenotyping’, may not align with MDD according to diagnostic criteria or represent associated but different genetic vulnerabilities^12–15^. In a comparison of depression phenotypes derived from the UK Biobank, Cai et al. (2020) found that minimal phenotyping definitions are epidemiologically distinct from strictly defined MDD, have lower SNP-based heritability (SNP-*h*^2^) estimates (11% for self-reported depression vs. 26% for MDD following diagnostic criteria), and that GWAS hits from minimal phenotyping may not be specific to MDD, and instead apply to a broader range of psychopathology and personality measures^15^. Consequently, follow-up characterization of GWAS loci from minimal phenotyping studies may not inform on biology specific to MDD.

The inclusion of data based on heterogeneous phenotypic assessment associates with a decay of SNP-*h*^2^ estimates^16,17^. This has led to a call for approaches with uniform phenotyping, for example by adherence to clinical diagnostic criteria. In addition to phenotypic heterogeneity, meta-analyses can introduce genetic heterogeneity. Nearly all current studies tend to limit participation to participants of European ancestry, but even here there can be considerable population stratification and admixture affecting heterogeneity and GWAS results^18^, even after adjustments through ancestry-informative principal components. Tropf et al.^19^ find that heterogeneity across populations is one of the contributing factors to missing heritability, the common difference between GWAS-derived SNP-*h*^2^ estimates and the upper limits set by twin-based approaches.

To address these challenges, we initiated the BIObanks Netherlands Internet Collaboration (BIONIC)^20^. This nation-wide collaborative project combines genotype data from 16 Dutch cohorts, and focuses on a phenotype definition of MDD according to DSM-5 criteria. The Netherlands has a population of 18 million people, with ~15.5 million of Dutch ancestry, and a population density of ~535 people per square kilometer (1,390 people/sq mi). In such single populations, population stratification is smaller, although not entirely absent^21,22^. We enriched the existing population cohorts and clinical cohorts in the Netherlands with an infrastructure for nation-wide depression phenotyping so that all MDD cases were defined based on DSM-5 (Diagnostic and Statistical Manual of Mental Disorders)-criteria^3,23^. All cohorts agreed to share genotype and phenotype data in a central location for mega-analysis, where QC and genotype imputation were conducted simultaneously and harmonized across all arrays and participants.

With the BIONIC dataset we ask the question if we can find evidence for a contribution of single nucleotide polymorphisms (SNP) to MDD as evidenced by genome-wide significant associations, significant SNP heritability and polygenic prediction. We report results from the mega-analysis of lifetime MDD case-control status and genome-wide SNP data in 64,941 individuals (N_case_ = 16,655, N_control_ = 48,286). We leverage clinical diagnostic criteria in a relatively homogeneous sample to identify genetic variant associations and consider the benefits of such an approach for capturing genetic signal, comparing our results to the largest GWAS of major depression to date (by the Psychiatric Genetics Consortium MDD workgroup)^10^. We benchmark these results with a genome-wide association mega-analysis of the well-characterized trait of height, for which a saturated map of common genetic variants has been generated^24^. We apply GWAS-by-subtraction, in which we searched for a uniquely Dutch MDD signal by ‘subtracting’ the largest global major depression GWAS effort from our GWAS results. We extend the GWA mega-analysis of MDD with a series of complementary analyses. These include fine-mapping approaches, such as gene-based tests and tissue expression analyses, as well as broader investigations into phenome-wide genetic correlations and causal relationships. We evaluate polygenic risk scores (PGS) through in- and out-of-sample predictions, assess potential confounding via within- and between-family tests, and examine twin concordance across PGS deciles.

## Results

### Genome-wide association mega-analysis of lifetime MDD

**Figure 1.**
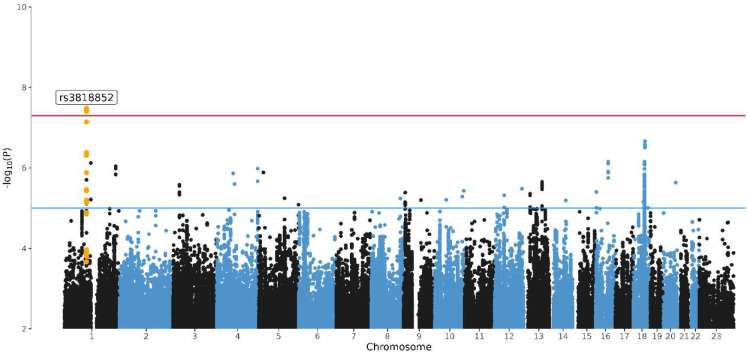
Manhattan plot of the GWAS mega-analysis of major depressive disorder. Manhattan Plot of the genome-wide association mega-analysis of lifetime MDD in BIONIC. The x-axis indicates chromosomal position. Y-axis denotes −log10 P-value and indicates the strength of association between the SNP and outcome phenotype. The red line indicates Bonferroni-corrected genome-wide significance; P < 5×10^−8^. The blue line indicates suggestive singificance; P < 1×10^−5^.

We ran a GWAS mega-analysis of lifetime MDD including 64,941 individuals (16,655 lifetime MDD cases and 48,286 screened controls). The majority of the sample was female (60.8% in the full sample) and average age was 50.7 (SD 16.0)(eTable 1). Figure 1 shows the Manhattan Plot of genetic variants and the significance of their association with lifetime MDD (QQplot in Supplementary Figure 2). The rs3818852 SNP on chromosome 1, an intronic variant of the PALMD gene, reached significance at the genome-wide-adjusted alpha level (OR = 0.93, p = 3.26 × 10^−8^)(regional association plot in Supplementary Figure 3). PALMD encodes the palmdelphin protein involved in cellular processes including cytoskeletal organization and cell shape modulation. There have been no previous GWAS hits for rs3818852 registered in the GWAS catalogue, indicating a potentially novel cross-domain phenotypic association. A second signal, rs4131791 on chromosome 18, showed suggestive significance (OR = 1.07, p = 2.17 × 10^−7^), and maps to the LINC03035 gene. Table 1 lists the 10 most significant independent SNPs, with the summary statistics for the top 1000 loci available in the Supplementary Material.

**Figure 2.**
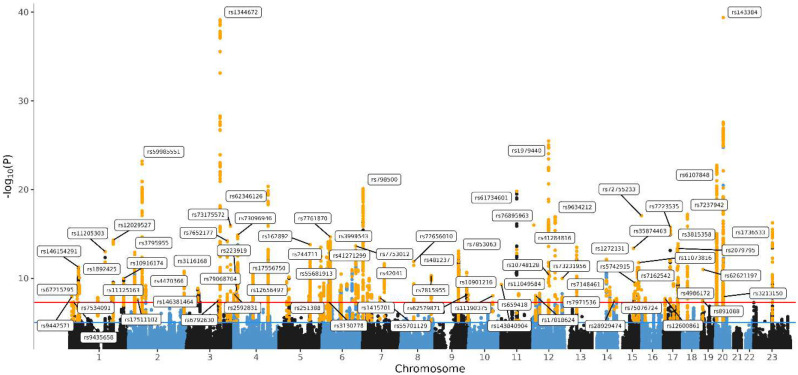
Manhattan Plot of GWAS mega-analysis of height. Manhattan Plot of the genome-wide association mega-analysis of height in BIONIC. The x-axis indicates chromosomal position. Y-axis denotes −log10 P-value and indicates the strength of association between the SNP and outcome phenotype. The red line indicates Bonferroni-corrected genome-wide significance; P < 5 × 10^−8^. The blue line indicates suggestive singificance; P < 5 × 10^−5^.

To study the feasibility of our analysis of MDD in this relatively small sample, we conducted a genome-wide association mega-analysis of height in 52,893 individuals with information on this phenotype from the BIONIC dataset (Figure 2, Supplementary Figure 4). Despite the modest sample size, we were able to identify 94 independent significant SNPs associated with height at the genome-wide significance level.

### SNP-based heritability and genetic correlation with PGC-MD

We ran Linkage Disequilibrium score regression (LDSC)^25^ on the GWA mega-analysis results of lifetime MDD, hereafter called the BIONIC MDD GWAS, to compute SNP-based heritability (SNP-*h*^2^). SNP-*h*^2^ for lifetime MDD in the effective sample size (N_eff_ = 63,742.64; N_cases_ = 16,655) was estimated to be 0.134 (se = 0.021) on the liability scale, suggesting 13.4% of phenotypic variance is jointly captured by the measured SNPs. The LD intercept estimate was 1.018 (se = 0.007), suggesting negligible inflation in GWAS test statistics due to sources other than polygenicity. We found a higher LDSC SNP-*h*^2^ point estimate than that in the largest GWAS to date, the Psychiatric Genetic Consortium meta-analysis of major depression (PGC-MD; SNP-*h*^2^ = 8.4%, se = 0.07%)^10^. This is consistent with evidence from previous studies showing that uniform phenotyping and clinical diagnostic criteria in a relatively homogeneous population benefit the capture of genetic signal of MDD^15,17^. We computed the genetic correlation between the BIONIC MDD GWAS and PGC-MD (European samples including 23andMe, N_eff_ = 1,523,738) in LDSC, finding a genetic correlation of r_G_ = 0.869 (se = 0.048).

Height LDSC SNP-*h*^2^ was estimated to be 0.379 (se = 0.026; N_eff_ = 49,036), or 37.9% of phenotypic variance. The genetic correlation with the largest GWAS of height to date, Yengo et al.^24^, was r_G_ = 0.980 (se = 0.016).

### GWAS-by-subtraction

**Figure 3.**
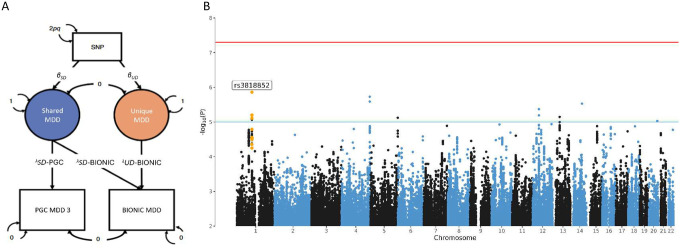
GWAS results of uniquely Dutch MDD (GWAS-by-subtraction). A) GWAS-by-subtraction model (Demange et al. (2021)). B) Manhattan Plot of the genome-wide association analysis of uniquely Dutch MDD, the latent factor resulting from subtracting the PGC-MD GWAS from the BIONIC MDD GWAS. The x-axis indicates chromosomal position. Y-axis denotes −log10 P-value and indicates the strength of association between the single-nucleotide polymorphism and the outcome phenotype. The red line indicates Bonferroni-corrected genome-wide significance; P < 5 × 10^−8^. The blue line indicates suggestive singificance; P < 5 × 10^−5^.

We investigated if a uniquely Dutch MDD signal might be identified through GWAS-by-subtraction^26^ (Figure 3A). The BIONIC MDD GWAS and PGC-MD GWAS were regressed on a latent factor representing genetic variance in depression aspects that are shared across the two studies, followed by the BIONIC MDD GWAS was further regressed on a latent factor representing genetic variance unique to the BIONIC MDD GWAS. This served as input for a GWAS of uniquely Dutch MDD (Figure 3B). In this GWAS, no SNPs reached significance at the genome-wide adjusted alpha level. However, the rs3818852 SNP on chromosome 1 that was found in the BIONIC MDD GWAS remained among the top signals (OR = 0.83, P-value = 1.38 × 10^−6^). LDSC SNP-*h*^2^ for this model was 0.036 (se = 0.014).

### Polygenic score prediction

**Figure 4.**
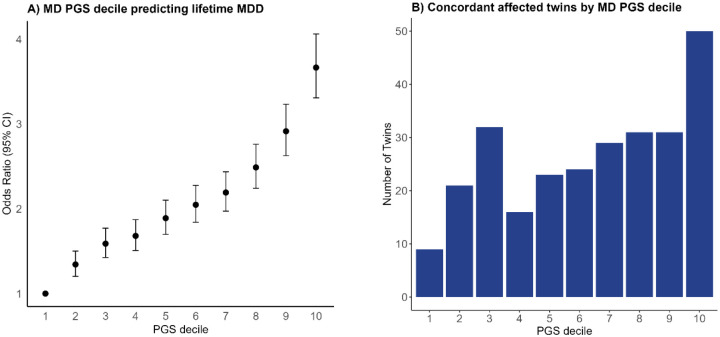
MD PGS prediction of lifetime MDD and twins by MDD PGS decile. A) Predictive PGS performance of MD PGS based on PGC-MD of lifetime MDD in BIONIC (unrelated N = 44,144). The x-axis denotes the MD PGS decile. The y-axis denotes the odds ratio of each decile from the generalized estimating equations model where lifetime MDD status in the BIONIC sample was predicted by PGS decile. Odds ratios were estimated relative to decile 1. 95% confidence intervals are given. B) Number of twins concordant for lifetime MDD status per MD PGS decile (N = 266 twins).

We tested predictive utility of the major depression polygenic score (MD PGS) based on PGC-MD in the BIONIC MDD GWAS sample (unrelated N = 44,144; 27% cases). The MD PGS was significantly associated with lifetime MDD status in BIONIC (OR = 1.054, *p* < 0.001), and explained 3.94% of variance on the liability scale. Figure 4A displays the PGS divided into deciles and their effect size.

We then tested the performance of a PGS based on the BIONIC MDD GWAS for two phenotypic definitions of depression in the UKBiobank: strict (ICD code for depression) and broad (‘minimal phenotypic’). The BIONIC MDD PGS was significantly associated with both strict (N = 23,755, OR = 1.163, Z = 17.1) and broad definitions of depression (N = 132,122, OR = 1.107, Z = 29.5), explaining 0.39% and 0.29% of phenotypic variance on the liability scale, respectively. These results highlight the transferability of the BIONIC MDD PGS, showing robust out-of-sample prediction for different phenotypic definitions.

### Within- and between-family PGS prediction

Genotype-phenotype associations and the PGSs derived from them may arise through confounding effects, including population stratification, assortative mating and gene-by-environment correlation^27^. To evaluate whether PGS prediction of lifetime MDD may be subject to confounding, we evaluated MD PGS performance based on PGC-MD in a subset of dizygotic twins from the BIONIC MDD GWAS sample. In an unmatched binary logistic regression model of 2282 dizygotic twins (1141 complete pairs), the major depression PGS significantly predicted lifetime MDD (OR = 1.434, *p* = 9.52 × 10^−9^). We then applied a random effects logistic model to predict lifetime MDD with between- and within-family MD PGS, and family as a random variable. Both the between- and within-family MD PGS were significantly associated with lifetime MDD (OR = 1.409, *p* = 9.04 × 10^−5^; OR = 1.948, *p* = 1.09 × 10^−6^, respectively). A chi-square test of the difference in between- and within-family MD PGS effects gave χ^2^ (1, *N* = 2282) = 3.983, *p* = 0.046. The between- and within-family PGS effects were significantly different, albeit barely so, suggesting that if there is confounding, it is minimal. Sensitivity analyses showed that the confounding may in part be due to sex-specific effects, as the difference for the between- and within-family MD PGS effects was strongest in sex-discordant twins (*p* = 0.025), but absent in sex-concordant twin pairs (*p* = 0.435)(Supplementary Information).

### Twin Concordance and PGS deciles

We computed MD PGS deciles in N = 2963 twin pairs in BIONIC. We distinguished three subsamples based on lifetime MDD concordance in the twin pairs: concordantly affected (N = 133 pairs), concordantly unaffected (N = 2257), and discordantly affected (N = 573). We expected concordantly affected twin pairs to be overrepresented in the high PGS deciles, and concordantly unaffected twin pairs to be overrepresented in the low PGS deciles. Figure 4B shows the distribution of concordantly affected twins across MD PGS deciles (concordantly unaffected and discordantly affected are shown in Supplementary Figure 5). Indeed, we found the number of concordantly affected twin pairs to increase in the higher MD PGS deciles, and the number of concordantly unaffected twin pairs to decrease in the higher MD PGS deciles. A similar pattern was observed in an independent replication sample from the Australian Genetics of Depression Study (Supplementary Information).

We ran an ordinal logistic regression model with twin MDD concordance as outcome to test whether the mean MD PGS predicted twin MDD concordance status in the N = 2963 twins in BIONIC. We found each increase of one standard deviation in mean MD PGS to be significantly associated with a 1.3-fold greater risk for concordant MDD status in twins (OR = 1.289; 95% CI = 1.174–1.404).

### Genetic causal associations

We computed genetic correlations between BIONIC lifetime MDD and 1461 complex traits and diseases, finding 388 significant genetic correlations (False Discovery Rate (FDR) < 5%) with well-established correlates including self-harm, substance use, anxiety, loneliness, neuroticism, irritable bowel syndrome, and chronic pain (Supplementary Material). With the latent causal variable (LCV) method^28,29^, we identified 33 traits with a putative causal genetic association with MDD at FDR < 5% (|GCP| > 0.60), and 4 traits with limited partial genetic causality (|GCP| ≤ 0.60)(Table 2; Figure 5). We identified 34 traits as putative risk factors for MDD, and 3 traits as putative outcomes of MDD. The putative causal genetic associations with MDD include a diverse range of traits, including well-known risk factors such as hypersomnia, stroke, and trauma. Several significant putative causal traits were related to one’s work environment, including working with paints and chemicals and workplace with very cold temperature, which might also reflect differences in socio-economic status. Others included lifestyle traits such as vitamin D levels and vegetable consumption. Finally, cardiometabolic traits were found, including stroke, ECG load, and HDL cholesterol.

### Gene-level analyses & tissue enrichment

We ran a MAGMA^30^ gene-based analysis on the BIONIC MDD GWAS results, mapping input SNPs to 19,210 protein coding genes. Two genes were significantly associated with MDD at the genome-wide significance level: the PALMD gene on chromosome 1 (Z-score = 4.725, P = 1.15 × 10^−6^) and the PIACIN1 gene on chromosome 16 (Z-score = 4.627, P = 1.85 × 10^−6^)(Figure 5). We ran MAGMA gene-set analysis for pathway-specific enrichment for gene sets C2 and C5 from MsigDB v7.0. We found no significant gene-sets at the Bonferroni-corrected significance threshold (3.23 × 10^−6^). The most significant gene sets were involved in regulation of T-cell migration (Beta = 1.61, P = 6.75 × 10^−6^) and vitamin binding (Beta = 0.31, P = 7.75 × 10^−6^). The full summary statistics of the gene-based test and gene set test are available in the Supplementary Material. In the MAGMA tissue expression analysis, in which we compared SNP associations from the BIONIC MDD GWAS with gene expression levels from the GTEx v8 database for 53 tissue types, none of the investigated tissues showed significant enrichment after multiple testing correction (Supplementary Figure 8).

### Gene prioritization

We applied FLAMES^31^ to find the most likely effector gene for the genome-wide significant locus in the BIONIC MDD GWAS (lead variant: rs3818852 on chromosome 1). There were 23 SNPs with an LD > 0.60 implicated as potential alternative causal SNPs, which were used for credible set generation in FINEMAP. The PALMD gene was assigned the highest FLAMES score (0.436), suggesting it is the most likely effector gene for the genetic association between rs3818852 and MDD.

### Sensitivity analyses

As an alternative to the GWAS mega-analysis approach, we conducted a genome-wide association fixed effect meta-analysis combining the seven array group GWAS results of lifetime MDD case-control status. Results were similar to those of the main model (r_G_ = 1, Supplementary Information). Further, we ran a GWAS mega-analysis of MDD in which the control group included individuals with evidence for psychopathology other than MDD to explore whether screening of controls led to biased SNP-*h*^2^ and r_G_ estimates. Total sample size increased to N = 67,440 (16,655 cases). The less stringent screening of controls had very little effect on the outcome (r_G_ = 0.99 with main model, Supplementary Information). SNP-*h*^2^ in this model was 0.118 (0.015), which is comparable to the first model (SNP-*h*^2^ = 0.134). This suggests the main model SNP-*h*^2^ estimate was not inflated by the screening of controls.

## Discussion

We ran a genome-wide association (GWAS) mega-analysis of lifetime major depressive disorder (MDD) in the Dutch BIONIC dataset, including MDD and genotype data on 64,941 participants. We created this resource to help unravel the complex genetic architecture of MDD by focusing on uniform phenotyping based on DSM-5 criteria in a genetically homogenous sample.

We found that common SNPs explained 13.4% of variance in lifetime MDD on the liability scale. This is a notable estimate compared to recent large-scale meta-analyses using a combination of broad and diagnostic definitions (8.4%)^9–11^. Though the proportion of variance explained by common SNPs can be subject to variation due to many aspects of study design, a notable contributor is heterogeneity in population, study design, and phenotype. Tropf et al.^19^ found that heterogeneity across populations was one of the sources for the difference between twin-based heritability and SNP-based heritability, the so-called missing heritability, and Cai et al.^15^ found that restricting samples to those meeting clinical criteria benefitted explained genetic variance. Our findings corroborate empirical evidence that both sample uniformity and phenotypic precision can enhance genetic signal detection^17,19^, joining other recent efforts that focus on this^32,33^. The success of our efforts reinforces the valuable contributions regional biobanks can have and suggests potential applications in studying other complex traits.

We report one genome-wide significant finding for MDD: rs3818852, an intronic variant of the PALMD gene on chromosome 1. To our knowledge, PALMD has not been implicated in MDD before. While its exact biological function is largely unknown, PALMD encodes the palmdelphin protein, which may be involved in cellular processes including cytoskeletal organization and cell shape modulation and is expressed in dendritic spines, membrane, and cytoplasm (NCBI, checked Feb 2025). Though rs3818852 has no previous associations, PALMD has been associated with complex traits from different phenotypic domains such as aortic valve calcification and the vaginal microbiome^34–36^. The A allele of the rs3818852 variant on chromosome 1 (PALMD) has a distinctly lower minor allele frequency (MAF) in BIONIC (0.379) and in the Genome of the Netherlands reference panel (0.374)^22^ compared to the global population (0.486, TOPMED, https://www.ncbi.nlm.nih.gov/, checked Feb 2025). Difference in allele frequencies has been proposed as the leading reason for the success of the CONVERGE study in detecting two of the first GWAS loci for MDD^37^, neither of which was significantly associated in GWAS on European samples^9,11^. The PALMD signal was further reiterated by the GWAS-by-subtraction analysis, in which the signal remained after subtracting the global depression signal captured by the international PGC-MD effort. The gene-based analysis identified two significantly associated genes with MDD, corroborating the PALMD and finding the CIAPIN1 gene. and found a significant association with the CIAPIN1 gene. CIAPIN1 is a protein coding cytokine-induced inhibitor of apoptosis involved in mitochondrial function, methyltransferase activity and iron-sulfur cluster binding. It has been found to inhibit hippocampal neuronal cell damage through MAPK and apoptotic signaling pathways^38^. To our knowledge, a role in MDD has not been established before.

Polygenic scores (PGSs) based on the BIONIC MDD GWAS significantly predicted distinct definitions of lifetime MDD in UKbiobank. The explained variance of the BIONIC GWAS PGS was higher for ICD10 diagnosis-based criteria, suggesting PGS based on strict phenotyping approaches may have higher transferability to other settings that use DSM criteria, such as the clinic. These results highlight that our PGS generalizes to other populations and phenotypic definitions. We also leveraged some of the unique features of the BIONIC dataset to explore polygenic score prediction of MDD in twin pairs. PGSs are increasingly becoming predictors and phenotypic proxies for phenotypes in genetic epidemiological designs^39^. GWAS and PGSs derived from these are naïve to the way in which genotype-phenotype associations arise, meaning they can reflect biological mechanisms but also may capture environmental factors, and thus may be subject to confounding^27,40^. By leveraging features of twin- and within-family designs, we examined confounding in PGS prediction of MDD, and found that if there was confounding due to population stratification, assortative mating and gene-environment correlation, it was minimal, corroborating findings from previous work where estimates of assortative mating in MDD were low^20,41,42^. We also explored the association between twin concordance and polygenic burden following a recent example in schizophrenia and bipolar disorder^43^. We show that twin concordance for MDD is partly a function of polygenic burden, with concordantly affected twins having a higher PGS than pairs where one twin was affected, who in turn had a higher PGS than concordantly unaffected twins.

Estimates of genetic correlations and genetic causal associations with other traits identified several putative risk factors and outcomes of MDD. We found hypersomnia to increase the risk of MDD, together with workplace-related traits such as a cold workplace, working with fumes and paint thinners, having had major operations, and cardiovascular indicators like stroke and triglyceride levels. We found other traits to reduce the likelihood of MDD, including vitamin D levels, HDL cholesterol levels, vegetable consumption and cod liver oil capsule consumption. MDD predicted that breathing problems improved when away from work, suggesting MDD may predict that breathing problems were caused by dissatisfaction or stress at work rather than an innate condition. MDD also predicted back pain to last over 3 months, and having been in a serious accident. In an earlier study of genetic causal associations with broad depression^29^, quite similar results were found, such as putative causal effects of hypersomnia, vitamin D levels, triglyceride levels, and various antidepressants. Conversely, the study found no putative outcomes of broad depression. It has been shown that minimally-defined depression includes nonspecific liability to psychopathology and that this affects genetic correlation estimates^15^. It is possible that this also affects genetic causal estimation estimates, and that considering clinically-defined MDD may reveal unique putative causal targets and outcomes.

There were several strengths and limitations. The sample size of this study was small compared to the standard of GWAS being published nowadays. Further, the results in this study were achieved by considering two factors, phenotypic homogeneity and population homogeneity. We cannot distinguish to which to attribute the increase in genetic signal, as indicated by the increase in SNP-*h*^2^. Finally, it cannot go without mention that the lack of diversity in the sample might hinder generalization of the findings to culturally-dependent phenotypic definitions and other ancestry-diverse populations. This study has been successful in recruiting cohorts that have not before been included in a GWAS of MDD, which we approached because they already had genotyping in the Dutch population available. We are not the first to show that a homogeneous and strict phenotyping definition benefits MDD genetic variant identification^37^. It is clear that such a strategy still holds promise for finding genome-wide significant associations with MDD in populations.

With this effort we add to the growing evidence base on the genetic architecture of MDD. We show that an effort with uniform phenotyping in homogeneous populations can aid genetic signal detection for MDD. As the genetic signal captured by broad phenotyping strategies plateaus, it may be time to invest in clinically-relevant phenotyping approaches, for which we provide a proof of concept.

## Online Methods

### Sample and phenotype descriptions

This genome-wide association (GWAS) mega-analysis combines major depressive disorder (MDD) and genotype data from sixteen Dutch cohorts in the BIONIC (BIObanks Netherlands Internet Collaboration) project. Descriptions of the sixteen cohorts can be found in the Supplementary Information. The majority of phenotype data were collected through the Lifetime Depression Assessment of Survey (LIDAS)^23^, developed to assess DSM-5 MDD. Other data were derived from clinical interviews and questionnaires (see eMethods for a detailed description). MDD cases were uniformly defined according to DSM-5 diagnostic criteria as ever having had a period of at least two weeks with five or more depression symptoms causing dysfunction in life, of which at least one a cardinal symptom^44^. MDD controls were defined as having no such period, and – when information on other psychopathology was available – having no other psychopathology (eMethods). The final analysis set included 64,941 European-ancestry individuals with non-missing MDD, covariate (sex, age) and genotype data, 16,655 lifetime MDD cases and 48,286 screened controls. Height data were available in 52,893 individuals.

### Genotyping, quality control, and imputation

Details on the quality control (QC) procedure have been described previously^20^. In brief, the sixteen cohorts had collected genotype data on seven different arrays across multiple batches, resulting in 19 datasets. This included some smaller datasets that could not be analyzed independently because they were too small or had an overrepresentation of cases. To include the largest number of individuals, SNP data genotyped on the same arrays were combined after basic sample and SNP quality control (details in eMethods). This resulted in seven array groups with raw genotype data from Affymetrix 6, Axiom Finngen, Axiom-NL, Illumina CytoSNP, Global Screening Array, Human Core Exome, and Omniexpress chip. These array groups were the units for further QC and imputation. Genotype data were imputed against the Human Reference Consortium (HRC) panel (v1.1)^45^. After imputation, the seven array groups had identical genome coverage and were merged into a single set for mega-analysis. All arrays included genotype data on the 22 autosomes. The availability of X-chromosome data varied (Supplementary Figure 1), with the first pseudo-autosomal region (PAR), non-PAR, and second PAR available in 55,141, 55,863, and 41,441 individuals, respectively.

We conducted principal component analysis (PCA) in PLINK (v1.9)^46^ on the imputed data based on the three superpopulations (African, Asian, European) from the 1000 Genomes Project reference panel (phase 3v5)^47,48^. The BIONIC genotype data were SNP and LD (linkage disequilibrium) pruned and projected onto the 1000 Genomes Project PCA space to compute principal components (PCs). Ancestry outliers in the BIONIC data were excluded based on a 4 SD distance from the mean of 0 for the first 6 standardized PCs. Additional ancestry outliers were removed based on visual inspection of PC clustering.

### Genome-wide association mega-analysis

We conducted a GWAS mega-analysis of lifetime MDD and height for SNPs on the merged seven array HRC-imputed dataset. Analyses were conducted in fastGWA^49^ from the Genome-wide Complex Trait Analysis (GCTA) software^50^, with a generalized linear mixed model for lifetime MDD and a mixed linear model for height. We computed a genetic relatedness matrix (GRM) in GCTA to address relatedness in the sample (sparse-GRM threshold 0.05). SNPs with a minor allele frequency below 0.01 and imputation quality below 0.40 were excluded from analysis. Analyses were corrected for sex, age, 10 ancestry-informative principal components and genotype array. Independent GWAS signals were identified by conditional and joint (COJO) analysis in GCTA^51^, where the BIONIC GWAS MDD sample served as the LD reference sample, restricted to one individual per related pair (relatedness ≥ 0.125) as calculated by the KING software (V2.2.6)^52^.

### SNP-based heritability and genetic correlation

The SNP-based heritability (SNP-*h*^2^) was estimated by Linkage Disequilibrium score regression (v1.0.1; LDSC)^25^. Effective N was calculated following previous literature^53^. For lifetime MDD, SNP-*h*^2^ was estimated on the liability scale, specifying 18% as population prevalence^3^ and sample prevalence as observed in the data (27% for the main GWAS model).

### GWAS-by-subtraction

To explore if there was evidence for specific Dutch MDD genetic signal, we carried out GWAS-by-subtraction as implemented in the genomic structural equation modeling (Genomic SEM)^54^ software with the GWAS summary statistics from the largest major depression (MD) GWAS to date^10^ (PGC-MD) and the BIONIC MDD GWAS as input. When applied to two traits, GWAS-by-subtraction performs a GWAS of the unique genetic variation, i.e. the variance not shared, between the two traits^26^. We defined unique Dutch MDD as the genetic variation in the BIONIC MDD GWAS that was not explained by the PGC-MD European GWAS (excluding the Netherlands), running a GWAS on the residual unique Dutch MDD genetic variation in the BIONIC MDD GWAS after the PGC-MD European GWAS was regressed out.

### Polygenic score prediction

We explored genetic risk prediction of lifetime MDD through in-sample and out-of-sample polygenic score (PGS) prediction. For in-sample prediction we generated MD PGSs in BIONIC based on the PGC-MD GWAS summary statistics (European samples and including 23andMe), excluding all datasets from the Netherlands^10^. PGSs were generated in the LDpred software (v0.9.1)^55^, a Bayesian method that derives posterior mean causal effect sizes from GWAS summary statistics by assuming a prior for the genetic architecture and linkage disequilibrium (LD) information. LD block information for West-European ancestry was derived from the UKBiobank^56^. The weights from the infinitesimal model were then used to perform allele scoring on the target sample with PLINK (v1.9)^46^. The association between the MD PGS and lifetime MDD case-control status in BIONIC was estimated by logistic regression in unrelated individuals with age at assessment, sex, and 10 ancestry informative PCs as covariates. The proportion of variance explained by the PGS on the liability scale for MDD case-control status was estimated according to Lee at al.^57^.

We explored out-of-sample MD PGS prediction based on the BIONIC MDD GWAS summary statistics in the UK Biobank, for a strict definition of MDD (clinical criteria) and broad depression (clinical criteria, sumscore cut-offs or self-report single item measures). Details on data field codes and genotype QC are in eMethods. A total of 23,755 and 132,122 individuals met the criteria for strict and broad phenotype definitions, respectively. PGS were generated in similar fashion to the in-sample prediction in LDpred (v1.0.10) and PLINK (v1.9) assuming an infinitesimal model. PGS prediction was estimated by logistic regression in unrelated individuals with age at measurement, sex, genotype array and 10 PCs as covariates.

Within-family designs are able to account for many sources of passive genotype-environment correlation, with dizygotic (DZ) twins having the additional benefit that all shared environmental factors are time-invariant among twins^27^. We sought to evaluate MD PGS prediction in a within-family design, following the approach of Selzam et al.^27^ with dizygotic twins from the Netherlands Twin Register (NTR; a collaborating cohort in BIONIC). We identified 1141 dizygotic twin pairs with non-missing lifetime MDD case-control status and MD PGSs. First, PGS prediction of MDD was assessed in the entire DZ twin sample through a logistic generalized linear mixed-effects model, correcting for sex and genotype array. Next, between-family PGS effects were defined as the change in lifetime MDD risk with a change in mean family PGS values, and within-family PGS effects as the change in lifetime MDD risk with a change in the difference between individual PGS and the family average PGS. The two were fit as predictors of MDD in the same model, together with a random effect for family, so that individual estimates are adjusted for and independent of the effect of the other estimate. The statistical difference between the between- and within-family PGS effects were then empirically tested through a chi-square test of the difference between their coefficients divided by the standard deviations of the sampling distribution of the estimate differences^58,59^.

Following a recent example in bipolar disorder and schizophrenia^43^, we computed MD PGS deciles in N = 2963 complete monozygotic and dizygotic twin pairs from the NTR cohort who took part in BIONIC. We then distinguished 3 groups: concordant affected (133) pairs, concordant unaffected (2257) pairs and discordant (573) pairs. We hypothesized that concordant affected twins would be overrepresented in high MD PGS deciles. We repeated this approach in an independent sample for replication, the Australian Genetics of Depression Study^60^ (Supplementary Information). We formally tested the association between the mean MD PGS in NTR twin pairs and MDD concordance in an ordinal logistic regression model, correcting for two ancestry-informative PCs^43^.

### Genetic causal associations

We computed genetic correlations between the BIONIC lifetime MDD GWA mega-analysis and 1461 disease, personality and lifestyle traits using bivariate LDSC in the Complex-Traits Genetics Virtual Lab (CTG-VL) analysis pipeline (https://vl.genoma.io/). Details on their derivation and acquisition are given in the eMethods. We applied the bivariate latent causal variable (LCV) model to the 388 traits from the CTG-VL pipeline with a significant genetic correlation with the BIONIC MDD GWAS. The LCV method identifies potentially causal relationships among heritable traits^28^, quantifying the degree to which a genetic correlation between two traits may be explained by (partial) genetic causality as opposed to full horizontal pleiotropy through the genetic causal proportion (GCP) metric. The LCV method assumes a genetic correlation between trait A and B is mediated by a latent variable (L) that represents the causal component between the two traits. GCP is based on the correlations between trait A and L, and trait B and L, and quantifies the proportion to which a genetic correlation can be explained by potential causal effects. Assuming no bi-directional causality, a GCP value of 0 suggests the genetic correlation is purely due to horizontal pleiotropic effects, whereas a |GCP| of 1 indicates complete genetic causality. Intermediate GCP values denote partial genetic causality, where some, but not all, of the genetic correlation can be explained by causal effects. A |GCP| > 0.60 is considered to be robust^61–63^. A negative GCP would suggest that a given trait is likely to be a risk or protective factor for MDD, whereas a positive GCP would suggest that MDD causes the trait. Benjamini-Hochberg’s False Discovery Rate < 5% was applied to correct for multiple testing at both the genetic correlation and LCV steps.

### Gene-level analyses & tissue enrichment

We performed a gene-based test and gene-set analysis of MDD in the MAGMA (Multi-marker Analysis of GenoMic Annotation; v1.08)^30^ tool. MAGMA employs a multiple linear principal components regression, and F test to obtain P values for genes^30^. In the gene-based test, input SNPs from the BIONIC MDD GWAS were mapped to 19,210 protein coding genes (symmetric gene window of 10kb), and tested for association with MDD in a SNP-wise top model. Pathway-specific enrichment of genetic signal for MDD was explored in competitive gene-set analysis with the gene-based analysis of MDD as input. Association between gene-set membership and gene Z-scores was computed using MAGMA. Gene sets were derived from categories C2 and C5 from the publicly-accessible MsigDB v7.0. Significance was set at the Bonferroni-corrected threshold of P = 0.05 / 15,488 = 3.23 × 10^−6^. We further performed tissue expression analysis in MAGMA with the gene-based test results as input. The MAGMA gene-property test compares average gene-expression for 53 tissue categories conditioning on average expression across all categories (one-sided) to test for tissue-specific enrichment. Gene expression datasets were obtained from GTEx v8 RNAseq. Significance was defined as P = 0.05 / 18,062 = 2.77 × 10^−6^.

### Gene prioritization

We fine-mapped the genetic association results in the Fine-mapped Locus Assessment Model of Effector geneS (FLAMES)^31^ software. FLAMES integrates machine learning predictions linking SNPs to genes with GWAS-wide convergence of gene interactions, to predict the most likely effector gene in a locus. Significant loci in the BIONIC MDD GWAS were subjected to FLAMES to determine the effector genes. Gene Z-scores and tissue enrichment estimates were derived from the MAGMA gene-based test and tissue expression analyses, respectively, and served as input for Polygenic Priority Score (PoPS; v0.2)^64^. An LD matrix was generated based on the BIONIC MDD GWAS sample in LDstore2 (v2)^65^ and posterior probabilities for 95% credible sets of causal SNPs were derived from the MDD GWAS summary statistics using FINEMAP (v1.4)^66^. Information from the MAGMA, PoPS and FINEMAP analyses were combined in FLAMES to generate the FLAMES score, an estimate between 0 and 1 assigned to genes near the associated locus where the highest estimate indicates the most likely effector gene. A cut-off FLAMES score of 0.05 was used to determine the putative effector gene in each locus.

## Supplementary Material

Supplementary Files

This is a list of supplementary les associated with this preprint. Click to download.

- BIONICGWASofMDDHuideretalsupplementaryinformation.docx
- BIONICGWASofMDDHuideretalsupplementarymaterial.xlsx

## Figures and Tables

**Figure 5.**
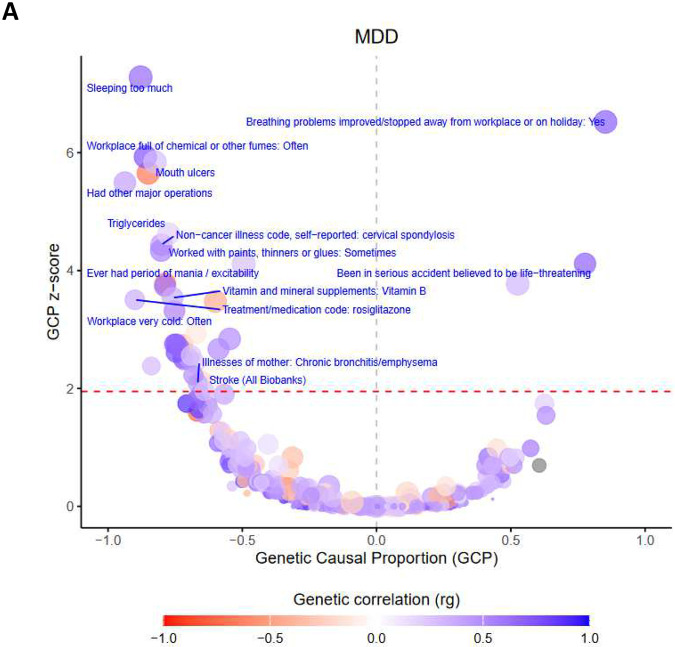

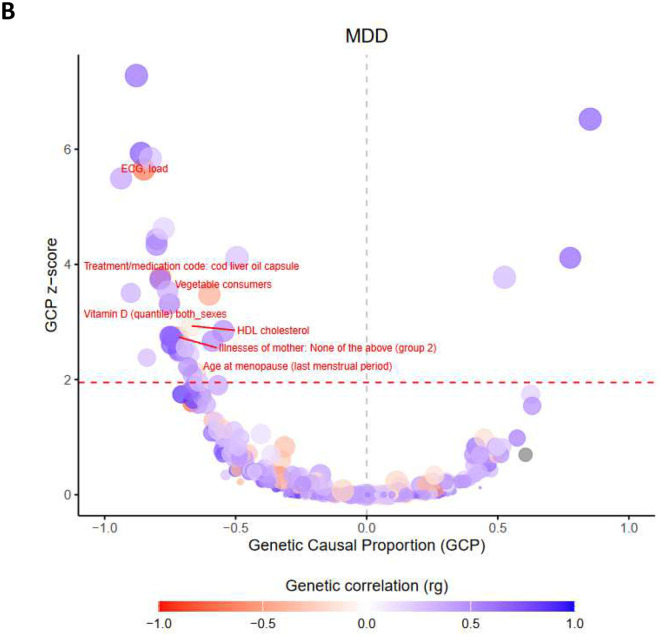
Causal architecture plots for MDD. Causal architecture plots depicting the LCV phenome-wide analysis results for BIONIC MDD. Dots represent traits with a significant genetic correlation with MDD. The x-axis reflects the proportion of genetic causality (GCP). The y-axis shows the GCP absolute Z-score (statistical significance). The red-dashed line represents the statistical significance threshold (FDR < 5%), while the gray-dashed line represents the division between traits causally influencing MDD (left) and traits causally being influenced by MDD (right). Dot size reflects estimate accuracy (se). Annotation is provided for traits with a |GCP| > 0.60 that are significant at False Discovery Rate (FDR) < 5%, and that have a positive (plot A) or negative (plot B) genetic correlation with MDD.

**Figure 5.**
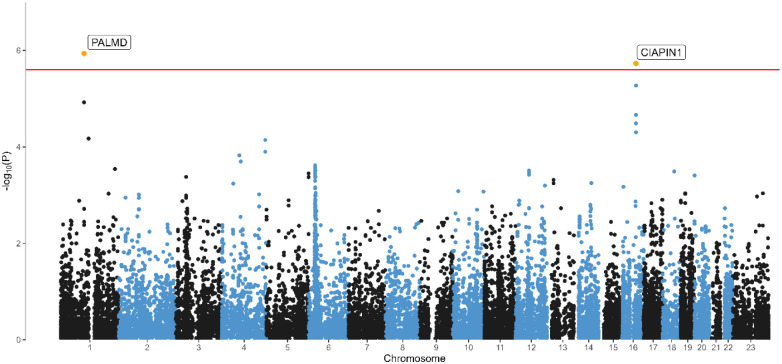
Gene-based analysis of MDD. Manhattan Plot of the gene-based analysis of lifetime MDD in BIONIC. The x-axis indicates chromosomal position. The y-axis denotes −log10 P-value and indicates the strength of association between the gene and the outcome phenotype. The red line indicates Bonferroni-corrected genome-wide significance; P < 2.50 × 10^−6^ (based on 19,994 tested protein-coding genes).

**Table 1.** Top 10 independent SNPs in the genome-wide association mega-analysis of lifetime MDD. Positions and rsID are in build GRCh17.

Chr	SNP	BP	A1/A2	F(A1)	OR	s.e.	*P*	Nearest Gene
**1**	**rs3818852**	**100160177**	**A/T**	**0.38**	**0.93**	**0.01**	**3.26e-8**	**PALMD**
18	rs4131791	52747871	C/T	0.58	1.07	0.02	2.17e-7	LINC3035
18	rs12327102	50787839	G/C	0.46	0.93	0.01	6.96e-6	DCC
16	rs62037142	57477652	C/T	0.98	0.76	0.04	6.97e-6	CIAPIN1
1	rs150207136	119607219	G/A	0.98	0.79	0.04	7.53e-6	WARS2
1	rs1970168	232761116	G/A	0.51	1.07	0.02	9.13e-6	SIPA1L2
4	rs10000918	186268576	A/G	0.74	1.08	0.02	1.04e-6	SNX25
5	rs33935044	21145325	A/T	0.98	1.32	0.08	1.30e-6	L0C102723561
4	rs138362425	75121348	C/A	0.98	1.35	0.09	1.37e-6	MTHFD2L
13	rs7491779	82485534	A/G	0.68	0.93	0.01	2.21e-6	None

Chromosome (chr), single-nucleotide polymorphism (SNP), base pair (BP), alleles (A1/A2; effect allele/non effect allele), effect allele frequency (F(A1)), effect allele odds ratio (OR), standard error (s.e.).

**Table 2.** Traits with an inferred putative causal relationship with MDD.

Phenotypes that cause MDD	GCP	GCP se	GCP *P*	r_G_	r_G_ se	r_G_ *P*
Sleeping too much	−0.88	0.11	8.64e-16	0.65	0.16	4.01e-05
Workplace often full of chemical or other fumes	−0.86	0.13	2.32e-11	0.73	0.19	7.56e-05
Mouth ulcers	−0.83	0.13	5.31e-11	0.28	0.07	4.74e-05
ECG load	−0.85	0.13	2.03e-10	−0.68	0.18	1.68e-04
Had other major operations	−0.94	0.15	6.11e-10	0.41	0.10	7.53e-05
Triglycerides	−0.77	0.14	6.77e-08	0.20	0.07	6.87e-03
Self-reported cervical spondylosis	−0.80	0.15	1.86e-07	0.44	0.15	2.38e-03
Sometimes worked with paints, thinners or glues	−0.80	0.16	3.31e-07	0.54	0.12	1.18e-05
Mouth-teeth dental problems: Mouth ulcers	−0.49	0.10	1.10e-06	0.28	0.08	2.49e-04
Treatment/medication code: cod liver oil capsule	−0.79	0.17	4.97e-06	−0.66	0.22	3.08e-03
Ever had period of mania / excitability	−0.79	0.18	6.68e-06	0.60	0.12	5.69e-07
Vitamin and mineral supplements: Vitamin B	−0.76	0.18	1.56e-05	0.36	0.13	6.57e-03
Treatment/medication code: rosiglitazone	−0.90	0.21	1.89e-05	0.35	0.14	1.20e-02
Vegetable consumption	−0.60	0.14	2.21e-05	−0.43	0.17	1.30e-02
Vitamin D	−0.75	0.18	4.35e-05	−0.18	0.06	1.97e-03
Workplace often very cold	−0.75	0.18	4.45e-05	0.51	0.15	4.61e-04
HDL cholesterol	−0.67	0.18	1.73e-04	−0.12	0.04	3.22e-03
Workplace often very hot	−0.55	0.15	2.43e-04	0.50	0.15	5.84e-04
Did your sleep change?	−0.75	0.21	3.44e-04	0.69	0.27	1.13e-02
Difficulty concentrating during worst depression	−0.75	0.21	3.47e-04	0.75	0.14	1.60e-07
Illnesses of mother: None of the above (group 2)	−0.72	0.20	3.75e-04	−0.41	0.12	1.12e-03
Workplace had a lot of cigarette smoke from other people smoking: Often	−0.74	0.21	4.57e-04	0.65	0.16	6.64e-05
More creative or having more ideas than usual	−0.59	0.17	5.10e-04	0.51	0.17	2.24e-03
Probable Recurrent major depression (moderate)	−0.75	0.22	6.30e-04	0.76	0.14	2.40e-08
Diabetes diagnosed by doctor	−0.69	0.20	7.56e-04	0.24	0.07	3.16e-04
Self-reported stroke	−0.72	0.22	8.71e-04	0.64	0.20	1.35e-03
Treatment/medication code: ranitidine	−0.70	0.21	9.20e-04	0.46	0.14	8.28e-04
Treatment/medication code: diazepam	−0.72	0.22	1.03e-03	0.63	0.24	1.00e-02
Self-reported diabetes	−0.68	0.21	1.23e-03	0.23	0.07	1.38e-03
Stroke (European biobanks)	−0.84	0.26	1.47e-03	0.30	0.11	8.61e-03
Diaphragmatic hernia	−0.68	0.22	2.30e-03	0.49	0.14	3.52e-04
Age at menopause (last menstrual period)	−0.65	0.22	2.99e-03	−0.22	0.06	7.67e-04
Illnesses of mother: Chronic bronchitis/emphysema	−0.67	0.23	3.42e-03	0.41	0.11	2.23e-04
Stroke (global biobanks)	−0.64	0.23	4.74e-03	0.27	0.09	2.94e-03
**Phenotypes caused by MDD**						
Breathing problems improved/stopped away from workplace or on holiday	0.85	0.12	3.55e-13	0.69	0.17	4.71e-05
Been in a serious accident believed to be life-threatening	0.78	0.16	1.08e-06	0.68	0.17	4.94e-05
Back pain for 3+ months	0.53	0.12	5.32e-06	0.31	0.11	6.39e-03

This table displays 37 traits with a significant (FDR < 5%) genetic causal proportion with MDD. P-values are before FDR correction, P-values after FDR correction are listed in Supplementary Material. GCP: genetic causal proportion; se: standard error; r_G_: genetic correlation.

## Data Availability

Data are available upon reasonable request from the contributing cohorts. Full summary statistics from the BIONIC GWA mega-analysis of MDD can be found here: https://tweelingenregister.vu.nl/bionic-mdd-2025
